# Socio-Sexual Experiences and Access to Healthcare Among Informal PrEP Users in the Netherlands

**DOI:** 10.1007/s10461-020-03085-9

**Published:** 2020-11-16

**Authors:** Mart van Dijk, John B. F. de Wit, Rebecca Kamps, Thomas E. Guadamuz, Joel E. Martinez, Kai J. Jonas

**Affiliations:** 1 Department of Work & Social Psychology, Maastricht University, PO Box 616, 6200 MD Maastricht, the Netherlands; 2 Department of Interdisciplinary Social Science, Utrecht University, Utrecht, the Netherlands; 3 Department of Society and Health, Mahidol University, Bangkok, Thailand; 4 Department of Psychology, Princeton University, Princeton, NJ USA

**Keywords:** HIV prevention, Informal PrEP, MSM, PrEP, Sexual behavior

## Abstract

The aim of this qualitative study was to explore the experiences of informal PrEP users regarding access to PrEP and PrEP-related healthcare, community responses, sexual behavior and well-being. We interviewed 30 men who have sex with men (MSM) in semi-structured online interviews between March and August 2018. Interviews were analyzed using interpretive description. Informal PrEP users were well informed about the use of PrEP, but sometimes did not make use of renal testing. Participants reported a lack of PrEP knowledge among healthcare providers, which limited their access to PrEP and put them at risk, as they received incorrect information. Although some participants reported negative reactions from potential sex partners, most received positive reactions and were sometimes seen as more desirable sex partners. PrEP healthcare services should not only be accessible to formal PrEP users, but also to PrEP users who procure PrEP informally.

## Introduction

Despite improvements in the availability of pre-exposure prophylaxis (PrEP), there is still a large gap between PrEP access and expressed need for PrEP in many countries around the world [1–3]. According to data from the European Centre for Disease Prevention and Control (ECDC) from 2019, formal access to PrEP is available only in 16 of 53 reporting countries in Europe and neighboring countries [4]. Access to PrEP has been limited due to structural barriers such as the high costs of PrEP, but also by a lack of PrEP implementation in existing healthcare settings [5–9]. As a consequence, some men who have sex with men (MSM) obtain PrEP via channels that have been labelled “informal”. These informal channels include online pharmacies, pharmacies and clinics in other countries where generic PrEP is sold at a cheaper price point, via post-exposure prophylaxis (PEP) treatments, and via pill sharing of HIV-positive people who take antiretroviral therapy [10–13]. In Europe, varying by country, up to 67% of PrEP users obtain PrEP via informal channels [4]. Informal PrEP use has been associated with suboptimal regimens and dosing of PrEP, a lack of knowledge about PrEP, and a lack of uptake of the recommended health services, such as regular HIV and renal testing and behavioral counseling [14, 15]. Inconsistent access to PrEP and PrEP-related health services, combined with the potentially suboptimal use of PrEP, puts informal PrEP users at risk for HIV infection and other health risks [15].

Most studies to date have investigated PrEP use in the context of clinical trials or in formal healthcare settings [16–25]. However, because the availability of formal PrEP healthcare is limited in many countries [4], there is a need to study PrEP use in such contexts of limited availability, reflecting a real world setting that for many imply informal use. Moreover, to optimize PrEP implementation, it is needed to understand how MSM comprehend and experience PrEP, thereby taking their psychological and social realities into account [26]. Informal PrEP use comes with specific challenges that cannot be observed in clinical trials, such as how PrEP is obtained and how to communicate with healthcare providers who are less informed about PrEP. So far, only a few studies have been conducted on informal PrEP use, mainly focusing on obtaining PrEP, using PrEP, and receiving medical support [11, 15, 27, 28]. While sexual risk behavior, including risk compensation, is often studied in the context of formal PrEP use [17–21, 23, 25], this is underexplored in the context of informal PrEP use.

While informal PrEP use typically refers to “non-prescribed” or “off-label” PrEP use [29], focusing on the procurement of PrEP, it is also important to consider how informal PrEP users access the appropriate medical supervision indicated with respect to PrEP use (i.e. CDC-guidelines [30]). A broader definition of informal PrEP use is hence the “non-prescribed and non-medically supervised” PrEP use [15]. This broader definition is particularly appropriate in the context of the Netherlands, and other countries where (generic) PrEP can be obtained before formal access is realized and formal PrEP-related medical supervision services are made available. The aim of this study was to explore the experiences of informal PrEP users in a context of the transition to full formal PrEP access. More specifically, we investigated the experiences of informal PrEP users across three domains: Access to informal PrEP and PrEP-related healthcare, and perceived community responses to PrEP use, and PrEP users’ sexual behavior and well-being.

### Access to PrEP and PrEP-Related Healthcare

Compared to the formal PrEP use, informal PrEP use may be challenging, as PrEP may not be easily obtained, and PrEP-related healthcare may often not be accessible. Obtaining PrEP and maintaining a consistent supply of PrEP is reported as a common difficulty for informal PrEP users [28], often resulting in the use of a regimen that requires less pills, for example an on demand regimen [31]. PrEP users may also be forced to take less pills than the recommended dosage. Nevertheless, a study among informal PrEP users in London found that despite difficulties in obtaining PrEP, most informal PrEP users had similar drug plasma concentrations as formal PrEP users in clinical trials [32]. In addition, informal PrEP users have to arrange the recommended counseling and three-monthly HIV, STI and renal function tests themselves, and they may also have to pay for these services out-of-pocket. Research has found that most of the informal PrEP users do not access PrEP-related healthcare, and do not regularly test for HIV [33]. Those who do access PrEP-related healthcare may be confronted with healthcare providers who may reject or stigmatize them for their informal use, or have a lack of knowledge or moralistic opinions about PrEP [34–36]. For example, healthcare providers were worried about risk compensating behavior [37, 38], and medical students were less likely to prescribe PrEP to patients with the highest risk behaviors [39], indicating that personal assessments of the healthcare provider play a role in the decision to prescribe PrEP or to offer medical supervision to PrEP users.

### Community Responses

PrEP users may face stigmatizing reactions regarding their PrEP use, and have been described in the media as “Truvada whores” [40, 41]. Among MSM, and even among PrEP users themselves, stigmatizing reactions are also noted [42]. For example, gay and bisexual men have been found to associate PrEP use with “barebacking” (intentional condomless anal intercourse), and to hold PrEP users responsible for the spread of STIs in the gay community [43]. PrEP users also report feeling stigmatized as a result of being rejected for sex dates or by being seen as HIV positive [44]. On the other hand, PrEP users can also be preferred as sex partners by MSM who are not using PrEP, as they are potentially at lower risk of transmitting HIV [45]. PrEP users have also been seen as more responsible and trustworthy, and their use of PrEP was not itself perceived as a reflection of sexual promiscuity [46]. Given these mixed findings regarding PrEP-related stigma, it is important to further explore the responses that informal PrEP users face from other MSM and potential sex partners. We did not expect differences in the perceptions of informal and formal PrEP users per se. It could however be that informal PrEP users are evaluated as having a poor “PrEP citizenship” [47] compared to formal PrEP users, as they may not have a reliable PrEP supply or are not being continuously monitored on HIV and STI testing.

### Sexual Behavior and Well-being

Sexual behaviors of PrEP users have mainly been studied in the context of formal PrEP use, in particular in the context of clinical trials and demonstration projects, such as ANRS-IPERGAY [48], AMPrEP [49], iPrEx [16], and PROUD [22]. However, clinical trials may give a biased view of changes in sexual behavior, because of the selection of participants (i.e. selected participants are MSM with (extreme) high risk behavior) or because of the included behavioral counseling, that may be absent in a real-world situation. Therefore, we also explored the sexual behavior of informal PrEP users in this study. A commonly reported change in sexual behavior after initiation of PrEP use is an increase in condomless anal intercourse [49]. Studies of formal PrEP use have also highlighted reduced fear of HIV, and improved sexual satisfaction and well-being [17, 50]. Similar findings have been obtained in studies of PrEP in non-clinical settings as well as in relation to informal PrEP use, including decreased condom use, reduced fear of HIV, and improved quality of sex life [27, 51, 52]. Informal PrEP use has also been associated with other increases in risk behavior, including recreational drugs use and group sex [11].

### The Current Study

The current study explored the behaviors and experiences of informal MSM PrEP users in the Netherlands. In the Netherlands, some MSM started to use PrEP informally before PrEP became formally available through Dutch pharmacies in 2016. Even after PrEP became formally available, informal procurement of PrEP continued, because PrEP was initially not affordable for many if not most MSM (~ € 500 per 30 pills). At the time, affordable PrEP in the Netherlands was only formally available through the AMPrEP study, a demonstration project that provided PrEP for free to 376 participants [19]. In November 2017, the price of PrEP in the Netherlands dropped significantly (to ~ € 50 per 30 pills) when generic versions became available in pharmacies in the Netherlands [53]. Although formal PrEP guidelines have been available in the Netherlands since 2016 [54], the practical implementation of PrEP was delayed as healthcare providers waited for an implementation policy of the Ministry of Health that addressed the provision of PrEP-related medical care. This PrEP implementation policy came into effect only in August 2019 [55, 56]. In other words, while PrEP users were able to obtain PrEP in the Netherlands as of 2016, it took another three years until arrangements of medical supervision of PrEP use were in place.

Investigating the experiences of informal PrEP users can inform healthcare professionals and community support groups about the challenges that informal PrEP users face. This investigation can provide guidance for necessary actions to provide PrEP related healthcare for informal PrEP users, and how to potentially bring them into formal PrEP care. Furthermore, our study also aims to corroborate previous findings of effects of formal PrEP use, such as on sexual well-being, as the type of procurement and use should not impact on the perceived benefit of PrEP.

## Methods

### Participants and Recruitment

In a survey that was conducted through the website of the Dutch PrEP advocacy group PrEPnu (www.prepnu.nl), 64 MSM indicated using PrEP informally. We invited these MSM for an interview, of which 26 (40.6%) agreed. In addition, four participants were recruited via peer referral, resulting in a total sample of *N* = 30. The participants did not receive any compensation for participation in the interview. The study was approved by the Ethical Review Committee of the Faculty of Psychology and Neuroscience, Maastricht University (code ERCPN 188_05_02_2018).

### Procedure

Between March and August 2018, 30 semi-structured interviews were conducted using an instant messaging program (Skype or WhatsApp, depending on the preference of the participant). We used instant messaging, because this allowed us to reach MSM from all over the country without being restricted by travel time and costs. In addition, because of the sensitive nature of the topics under investigation, we expected that MSM would express themselves more freely in an online chat interview compared to a (online) face-to-face interview, as anonymity can be ensured. Instant messaging has been suggested to be beneficial and fitting to study sensitive research questions, and produces comparable results as face-to-face interviews [57, 58]. Two interviews were carried out in English and 28 in Dutch. All participants provided informed consent at the start of the interview.

We developed an interview guide with fifteen questions, related to the topics of PrEP use, PrEP-related healthcare, PrEP communication, and sexual risk behavior. Every question was copied (one by one) into the chat window from the interview guide. Interviews were held in a conversational style and additional questions were asked for clarification or elaboration. The order of questions was adapted to the flow of the conversation. The interviews took a maximum of two hours and were conducted uninterruptedly.

Twenty-six interviews were conducted by a female interviewer (RK, 23 years old, master student in health and social psychology) and the remaining four interviews were conducted by a male interviewer (MvD, 28 years old, PhD student in applied social psychology). The first two interviews were jointly conducted by the interviewers, to align the use of the interview guide, and to ensure consistency and quality of the subsequent interviews.

### Data Analysis

The experiences of informal PrEP users were explored using interpretive description [59]. The data was sorted into categories in line with the topics of the interview guide, and we identified and described issues that emerged during the interviews. The interviews were coded in a deductive and iterative process, using the qualitative data analysis software ATLAS.ti version 8.3.0 [60]. The first five interviews were coded by the two researchers independently. The researchers discussed the coding to improve reliability of their interpretation. Inconsistencies in coding were resolved through discussion. Thereafter, the remaining interviews were coded by only one of two researchers (23 by RK and 2 by MvD). After coding, the results were saved in a summarized manner in an Excel table for further analysis. Quotes that were originally in Dutch were translated verbatim into English, when reported in the results section.

## Results

### Participant Characteristics and PrEP Use

The age of the participants ranged from 24 to 66 years; about half of the participants (40.0%) were younger than 40 years. The participants resided all over the Netherlands: Most of the participants lived in an urban area (12 in Amsterdam, 11 in other cities in the Netherlands), and seven participants lived in a rural area of the Netherlands. Eighteen participants (60.0%) were single and 12 participants (40.0%) were in an open relationship. Nineteen participants (63.3%) used PrEP in a daily regimen and 11 participants (36.7%) used PrEP on demand. The average time since commencement of PrEP use was 15 months (range: 3–60 months).

### Methods of PrEP Procurement

Most participants obtained PrEP abroad, notably in Thailand, India, and South-Africa, where PrEP at the time (2015–2017) was available for a lower price than in the Netherlands (approximately € 30 per 30 pills). They travelled to these countries for their jobs or for holidays, or they asked friends to bring PrEP from these countries. A minority also bought PrEP online from sources abroad, although some MSM experienced that their shipment was intercepted by customs in the Netherlands, or that the shipment took long to arrive. As soon as cheaper generic versions of PrEP became available in the Netherlands (November 2017), most participants switched to obtaining PrEP via local pharmacies, as it was faster and cheaper than buying PrEP abroad. However, some participants continued to obtain PrEP from sources abroad, as they reported to be used to the procedure, or found it more convenient since a friend obtained it for them, as illustrated by one participant:

“…because I am used to this way [of obtaining PrEP], and I don’t have to do much effort for it, thus simply out of convenience.” (P21)

### Challenges in Access to PrEP-Related Healthcare Services

In the Netherlands, the healthcare services related to PrEP can be accessed via general practitioners (GP), medical specialists, pharmacies, and the public health centers for HIV/STI testing. Almost all participants received a prescription from their GP, which allowed them to buy PrEP in a pharmacy abroad or online, and later also in the Netherlands. Most of them were satisfied with their GP regarding PrEP care. Some participants perceived a lack of knowledge among their GPs about the use and effectiveness of PrEP, or even a lack of awareness. Nine of these participants indicated that their GP took time to learn about PrEP, for example by consulting a specialist in the hospital or a public health center. Moreover, participants themselves could be a source of information for their GPs, and provided PrEP information to their GPs:

“We [my partner and I] have a very open dialogue with our GP. He knows that we have an open relationship and he has always supported us in the use of PrEP. In fact, I am his informant when it comes to the latest information. He applauded that we take responsibility ourselves regarding safe sex.” (P4)

It is noteworthy that some participants had selected their GP based on the gay friendliness of the GP, sometimes already before obtaining PrEP, which made it easier to discuss PrEP with their GP. At least three participants switched GPs when they wanted to obtain a PrEP prescription, as their previous GP did not want to support their needs:

“I had to switch GPs, as the previous one had very old-fashioned ideas about sex and numbers of partners. The one I have now is understanding, thinks along, thinks about alternatives, and in case of STIs he asks questions in a professional way.” (P3)

**Fig. 1:**
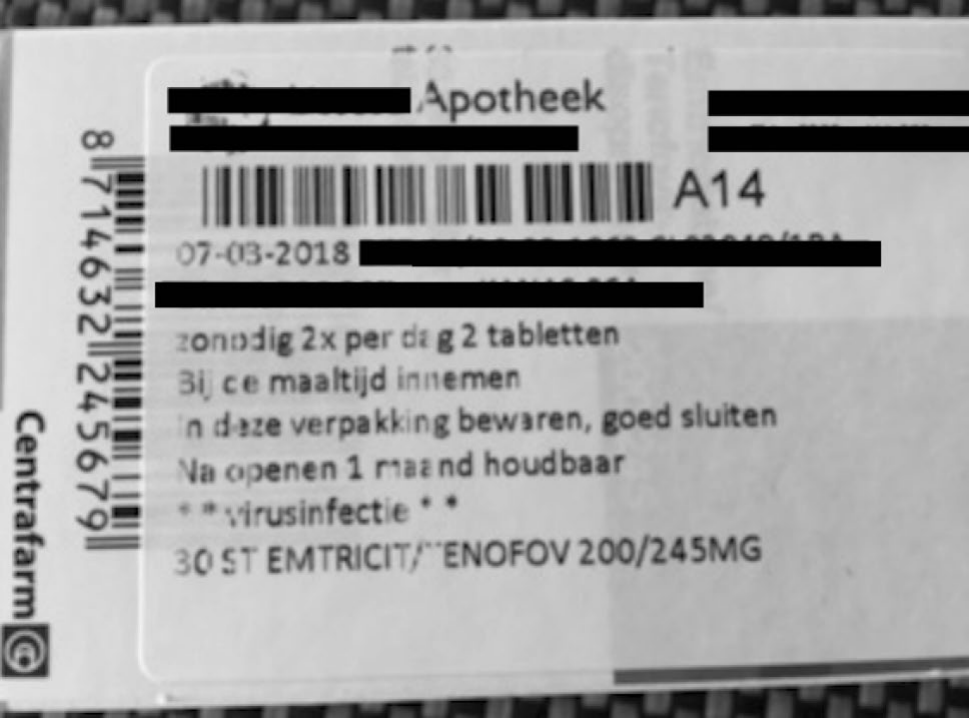
Image provided by an interview participant. Label on the package of generic PrEP pills of one of the participants, stating the misinformation regarding dosing scheme and the use for the treatment of a virus infection. Translated from Dutch: “Date: 07–03-2018. If necessary, 2 times per day 2 pills. Take with meals. Store in this package, close well. After opening one month shelf-life. **virus infection**”. Please note that the correct PrEP dosing scheme is one pill per day for a daily regimen, and that PrEP is meant for the prevention instead of treatment of a virus infection

After receiving the prescription from the GP, people can obtain PrEP in their pharmacy (note that in the Netherlands, clients are usually registered with a specific pharmacy). Six participants stated that their pharmacy had sufficient information about PrEP, whereas others stated that their pharmacy did not know a lot about PrEP. For example, the pharmacist assumed that they were HIV-positive and mistook PrEP for antiretroviral treatment (ART), or mistook PrEP for post-exposure prophylaxis (PEP). In one case made known to us, this lack of knowledge amongst pharmacists resulted in putting misinformation and unclear information on the PrEP package, including an incorrect dosing scheme, and only mentioning “virus infection” without any reference to prevention (Fig. 1). This puts PrEP users at risk of wrong dosage, and may lead to the misperception that the user is HIV positive, which could create social barriers to use PrEP:

“[The label on] the jar always states *virus infection*. I do not like that, because I just place it at home on the kitchen table. If you have people visiting, they immediately think you are sick.” (P31)

This unclear, and sometimes erroneous, information about PrEP use from healthcare providers puts informal PrEP users at risk for incorrect PrEP use. It places a burden on PrEP users to be well informed about PrEP themselves to be able to educate their healthcare provider.

After obtaining PrEP, informal PrEP users can arrange regular HIV and STI testing at the public health centers (for free) or via the GP. Renal function testing can be done via the GP, but must be paid for by the client up to a maximum of € 385 per year. Almost all participants reported that they underwent HIV and STI testing every three months. Renal function testing was done less frequent and by less participants compared to HIV and STI testing. A few participants did not do renal function testing at all, because they thought it was not necessary as they were using PrEP on demand, or because it was too expensive. This lack of renal testing demonstrates that while informal PrEP users often have a prescription for PrEP, medical supervision is not always present. Most participants had found a testing routine that worked well for them, with (free) STI testing carried out in public health centers and (paid) renal function testing done via their GP, although some participants reported difficulties with finding test locations for renal testing:

“[I do] not [test my renal function]. But I use [PrEP] sporadically, and your renal function should recover when you stop using the medication. I would like to test [renal function] more often, but I do not know how. At the STI clinic, where I come twice per year, they do not offer that.” (P20)

Some participants also reported difficulties with making an appointment in public health centers as they faced waiting lists of more than four months for HIV and STI testing. Participants who lived in multiple places also mentioned that PrEP-related healthcare is more developed in Amsterdam compared to other places in the Netherlands:

“In the big cities it works. The rural area and the villages… for that matter the PrEP care is dependent on where you live, and that is worrisome.” (P1)

To summarize, themes in challenges of informal users arranging their own access to PrEP-related healthcare services were gay-friendliness of GPs, lack of information and knowledge among GPs and pharmacists, and availability and pricing of testing facilities for HIV and STI testing as well as renal function testing.

### Community Responses: PrEP Related Reactions from Sex Partners and Friends

Some participants did not disclose their PrEP use to (potential) sex partners, because they did not find it necessary to discuss this with the partner, as they saw PrEP as a preventative measure for themselves, independent of the other person. When participants disclosed their PrEP use, about half of them experienced negative reactions, especially in online dating apps. For example, they got rejected for a sex date, or they were seen as more promiscuous. On the other hand, most of the time participants experienced neutral or positive reactions by other MSM. For example, they were seen as taking care of their health. Moreover, informal PrEP users received questions on dating apps from other MSM who were interested in PrEP. They received questions about how to obtain PrEP and where to buy it.

Participants mentioned that other MSM assumed that PrEP users only want condomless sex, which was seen as both favorably and unfavorably by sex partners: Favorably because the other MSM preferred to have condomless sex and believed a PrEP user would agree on this; unfavorably because the other MSM associated PrEP use and condomless sex with irresponsibility:

“There are people who did not want to have sex with me, because I used PrEP and thus I was seen as irresponsible. This happens mainly with younger guys [16–24] and it happens approximately once every two months. I try to let these young guys realize that they also have a chance to get HIV, and this chance is much higher [when they have sex] with someone who does not use PrEP than with someone who uses PrEP.” (P12)

When participants encountered negative reactions from other MSM on online dating apps, they noticed that this was sometimes a reflection of a lack of knowledge about PrEP. This was especially the case for participants from more rural areas or in the “early years” of PrEP use (2014–2015). As a result, PrEP users often found themselves educating other MSM about PrEP:

“Occasionally [I get] a negative reaction. But that is because they do not understand what it is and what it does. Then I explain it properly.[…] It arises often from ignorance, they think for example that you have HIV. Or they form a notion of that you are a dirty slut.” (P14)

Some participants told their gay friends about their PrEP use and received mainly positive reactions. One participant mentioned that PrEP helped to raise issues of HIV and STI with gay friends. In addition, some participants mentioned that gay friends started PrEP use after discussing PrEP and sexual health:

“And also with gay friends, I have had more conversations about STI / HIV in the past six months than ever before. It feels really like a kind of smokescreen has disappeared. More and more [friends] use it. At least among my friends there is a lot of discussion about [PrEP].” (P30)

The fact that PrEP was obtained informally did not stop others to want starting to use PrEP too. It helped informal PrEP users to connect socially, and one participant mentioned that he ordered PrEP in Thailand for his friends too.

Some participants also told their family and heterosexual friends about their PrEP use. Family and friends were surprised that something like PrEP existed, and were proud or jealous of how participants managed their sex life. The family of one participant was worried about his health and if PrEP (without condoms) would be enough protection for him. At least two participants did not tell their family about PrEP because they were ashamed to tell or feared to be judged.

### Changes in Sexual Behavior and Sexual Well-being

Despite the informal way that these participants obtain their PrEP, they experienced similar responses to using PrEP as those reported by formal PrEP users. Most participants (*N* = 22) reported a decrease in condom use since they started using PrEP. For some it was a deliberate decision to stop or decrease condom use when on PrEP, because they had erection problems when using condoms or they doubted the effectiveness of condoms. For others, condom use decreased because they felt less discipline to use condoms consistently. Some participants emphasized that it would not make sense for them to use both condoms and PrEP:

“During a PrEP information meeting two years ago, it was said that it is the intention that you use PrEP *and* condoms together. I thought that was nonsense at the time. Why use heavy medication if you still have to use a condom?” (P20)

Although there was a decrease in condom use for most PrEP users, there were situations in which most participants continued to use condoms. This was the case when the sex partner requested to use condoms, or when the participants assessed the sexual encounter as more risky. They evaluated this risk based on the level of familiarity with the sex partner, his PrEP use, and his assumed number of other sex partners:

“With a stranger I always do it safely, so with a condom. With a number of fuckbuddies I have unprotected sex. These are people who also use PrEP and I know that they do not have many changing contacts.” (P4)

Changes in sexual behavior were related to improvements in sexual well-being. Most participants enjoyed sex without condoms more, and it contributed to a perceived increase in the quality of their sex life. Sex felt more unrestrained and intimate, and they had less worries during sex:

“For me a lot has changed [with anal intercourse]. It has mainly to do with condom use.[With condoms] it feels literally like there is no contact. When I am bottom, it is more painful. When I am top, I awfully quickly ejaculate. And a condom feels very unpleasant.” – *Interviewer*: “So the quality of your sex life improved enormously?”—*P7*: “Enormous.” (P7)

The increased quality of their sex life was also reflected in changes in sexual experience. Participants felt less worried about getting HIV, and this resulted in increases in sexual activity and less concerns about who to have sex with. They also reported being able to enact fantasies that they did not dare to before using PrEP. For example, some participants reported that they felt more comfortable now with being the receptive partner (bottom) during anal intercourse:

“I also notice that I now dare to give in to fantasies that I have always been cautious about.[…] I also have sex with more men now.[…] Now I can do things that I like. Before, I found it scarier to be fucked in the passive role. Even with a condom. Does it slide off, or would someone take it off without a word? I was more preoccupied with these control thoughts, while one wants to indulge in sex. These thoughts restrained me in experiencing sex spontaneously.” (P5)

For some participants the use of PrEP felt liberating, mostly because they felt less fear of HIV. About half of the participants reported memories of the early days AIDS, and some of them reported that they always associated sex with fear of HIV, which limited their sexual pleasure:

“Every morning after having sex with a guy, I start thinking about whether there has been a risk of HIV. So instead of feeling comfortable [with having sex with a guy], the fear dominates. Since I take PrEP, the fear has been gone.[…] Gay sex and HIV are almost directly related to each other.[…] Instead of sex being something pleasant, it is something dangerous for gays because one can run into a serious problem.” (P17)

Also younger participants, who became sexually active in a time when HIV was a manageable condition, reported fear of HIV, which was reduced by the use of PrEP:

“Before I used PrEP, I sometimes could not sleep well, and I regretted often having sex. Also I could not really enjoy sex fully. I was aware that I would ever get HIV, the only question was when.” (P12)

For others, condomless sex itself felt liberating. After many years of using condoms, they felt hesitation about not using condoms, but they got easily accustomed to it:

“It took me some effort to accept the idea that sex without a condom was also “safe” for HIV. But after having bare sex with my regular sex partner, it was such a liberation that I was soon over it.” (P28)

About half of the participants reported using recreational drugs during sex. Most of them already used drugs before they started using PrEP. For some participants drug use increased, as it often co-occurred with going to sex parties. PrEP was beneficial for them as they had to worry less about condomless sex:

“I mostly use XTC [ecstasy/MDMA] and GHB [when going to sex parties]. I do not use it always, but when I do, the use of PrEP is nice, because you do not always know afterwards what happened.[The use of PrEP and drugs] are independent of each other. But the use of PrEP has many advantages, I think. I do not have to worry about how the sex is being done.” (P6)

## Discussion

The aim of this study was to explore the experiences of informal PrEP users in a context where PrEP is formally available, but formal implementation is still limited. We in particular explored the experiences of informal PrEP users across three domains: Access to PrEP and PrEP-related healthcare, community responses, and sexual behavior and well-being.

### Access to PrEP and PrEP-Related Healthcare

We found that informal PrEP users have found their own ways and routines in accessing PrEP and PrEP-related healthcare. Many of our participants switched from obtaining PrEP abroad to obtaining PrEP via local pharmacies in the Netherlands, as soon as generic PrEP became more affordable in the Netherlands. In contrast with earlier studies that found that informal PrEP users are less likely to undergo frequent HIV and STI testing [14, 15, 33], most participants in our interviews tested regularly. While this paints a positive picture of the uptake of PrEP healthcare services among informal PrEP users, findings also identify several major concerns.

Firstly, GPs, who play a critical role as gatekeepers in the Dutch healthcare system, were not always able or willing to support the needs of informal PrEP users. The participants in our study were well-informed about PrEP, and that helped them to either educate their GPs, or to change to another GP that did support their needs. MSM who are less informed about PrEP may however not be able to get the support they need from their GP, which would preclude them from accessing PrEP, or could force them to obtain PrEP via informal channels.

Secondly, some participants experienced long waiting lists for HIV and STI testing at public health clinics. While this not only puts PrEP users at risk of having and transmitting an undetected STI, it may also result in them doing less testing, not testing, or discontinuing PrEP use [61], which eventually may increase their risk of HIV.

Thirdly, renal function testing was not always optimal, as was also found before among informal PrEP users in Germany [31]. Similar to the “DIY PrEP” study [27], we found that some PrEP users find renal function testing not always necessary, but also that some PrEP users did not know where to go for these tests, as it was not available in the public health clinics where HIV and STI testing are offered. In addition to setting up adequate PrEP consultations to inform MSM about the use and potential side effects of PrEP, a convenient testing location should be organized where informal and formal PrEP users can go for all the necessary tests on a regular basis.

### Community Responses

PrEP users mostly got positive reactions. For example, they were seen as more desirable sex partners. It has been suggested that PrEP users are more likely to be seen as HIV-negative, and therefore have a lower chance to transmit HIV, making them a more desirable sex partner [45, 62]. While media outlets can present PrEP use in a stigmatizing way (i.e. “Truvada whores” [40, 41]), and stigmatizing reactions even occur among PrEP users themselves [42], our study provides a more nuanced view on how informal PrEP users are seen by community members in which general PrEP knowledge is emerging.

PrEP users nevertheless encountered negative, or stigmatizing, reactions. This included being rejected by a potential sex partner, or receiving negative comments in dating apps. Negative responses were found to be especially common in the early years of PrEP availability in the Netherlands and in rural areas, suggesting that unfamiliarity with PrEP may lead to negative responses. The informal PrEP users in this study were able to deal with negative reactions, and were able to educate other MSM about their PrEP use, because they were well-informed. Especially in countries where PrEP is not formally implemented, MSM are more likely to be informed about PrEP via peers, and they may find it challenging to obtain reliable information.

### Sexual Behavior and Well-being

Most informal PrEP users reported an increased quality of their sex life, mainly driven by less fear for HIV infection and decreased condom use. Although it is not surprising that condom used decreased, and this has been found before [63], it is an important finding that condom use was a prominent topic in many of the interviews. It often played a role in participants’ considerations on how PrEP increased the quality of their sex life, as PrEP use helped participants to explore ways of having sex, for example by switching sexual positions (insertive/receptive) or by experimenting with substance use and participating in sex parties. This is in line with findings from other countries and in earlier studies [17, 27, 50, 64], and seems to reflect an overall trend, regardless of whether the type of procurement and use is informal or formal. Participants noted that the reduced fear for HIV felt liberating and improved their sex life. The sexual liberation related to PrEP use has been found before [50, 65], and indicates that PrEP users feel more in control of their sex life [66].

### Continuing Informal PrEP Use or Moving Towards Formal PrEP

In our study we found that many PrEP users switched from the informal procurement of PrEP to a formal procurement of PrEP in local pharmacies. At the same time, PrEP implementation programs were not in place yet, so PrEP-related healthcare, in particular counseling, and regular HIV, STI and renal function testing was not formally accessible for them. The participants in our study had to organize this themselves, and most were quite well able to do so.

Informal PrEP procurement may continue to exist, even when the formal accessibility of PrEP is improved. On a personal level, MSM might prefer to continue obtaining PrEP via informal channels, as one participant mentioned in our study that he did this out of convenience. A study in Germany found that, despite the formal availability of PrEP, 17.4% of PrEP users continued to obtain PrEP informally, mainly because the price of generic PrEP (€ 50 per month) was still unaffordable to them [31]. On a structural level, there are many countries in the world where no steps are taken (yet) to make PrEP available (see https://www.prepwatch.org/ for country updates, and for Europe the ECDC report [4]), so for many (potential) PrEP users around the world there are no opportunities yet to formally access PrEP use.

We found that even when participants switched to formal PrEP procurement, formal PrEP-related healthcare was not always readily accessible. To facilitate a transition into formal PrEP-related healthcare, it is important to consider the needs of PrEP users. The PrEP-related healthcare service should provide one-stop access to counseling, renal function testing, and HIV and STI testing. The service should not only be affordable, but also convenient and non-judgmental. There are already several initiatives to offer these services to informal PrEP users, such as the InPrEP project in Amsterdam [67] and CheckpointLX in Lisbon, Portugal [68].

### Limitations

Several limitations of this study should be noted. We recruited participants via a survey that was posted on the advocacy website PrEPnu.nl, which provides information on accessing and using PrEP. This means that our participants had access to extensive information on PrEP. It is also important to note that the informal PrEP users in this study can be considered innovators or early adopters of PrEP, who had to be well-informed in order to be able to obtain PrEP. Their knowledge also allowed some of them to convince their GP to prescribe PrEP, and to arrange regular HIV and STI testing themselves. The recruitment and sample characteristics may limit the generalizability of the results of our study.

Furthermore, we used online chat messaging to conduct the interviews. While this may lead to less detailed responses [69], the results are overall comparable to those of studies based on face-to-face interviews [57, 58]. The interviewers were specifically instructed to invite participants to elaborate, in particular regarding their emotions or feelings. Nevertheless, some participants kept their answers rather short. We believe that online chatting is a valuable data collection tool, as it facilitates anonymous interaction. Moreover, in the context of e-health interventions it is not uncommon for MSM to discuss PrEP and sexual behavior online [70]. This makes the transition to discuss these topics in an online chat interview relatively easy.

## Conclusions

This study highlighted the experiences and behaviors of informal PrEP users in a context of transition to full formal PrEP healthcare implementation. Overall, informal PrEP users were doing quite well regarding PrEP use and accessing PrEP-related healthcare. PrEP healthcare services should not only be accessible to formal PrEP users, but also to PrEP users who procure PrEP informally. Global and national guidelines may be helpful to implement these services, but as the example of the Netherlands in this study shows, the availability of national guidelines alone does not directly make practice. Informal PrEP users still have to take the initiative to inform their healthcare providers about their self-medication, which may be frowned upon, and bears the risk of not receiving the necessary care due to avoiding disclosure of PrEP use. The experiences of informal PrEP users regarding community responses and sexual behavior were in line with findings of studies of PrEP use in a formal context. The findings of our study are not only relevant for the Netherlands, but also for other high-income countries where formal PrEP is not implemented yet.
